# Early Enteral Feeding of the Preterm Infant—Delay until Own Mother’s Breastmilk Becomes Available? (Israel, 2012–2017)

**DOI:** 10.3390/nu14235035

**Published:** 2022-11-26

**Authors:** Noa Ofek Shlomai, Yonatan Shneor Patt, Yaara Wazana, Tomer Ziv-Baran, Tzipora Strauss, Iris Morag

**Affiliations:** 1 Department of Neonatology, Hadassah Medical Center, Faculty of Medicine, Hebrew University of Jerusalem, Jerusalem 9190501, Israel; 2 Sackler Faculty of Medicine, Tel Aviv University, Tel-Aviv 6997801, Israel; 3 Department of Epidemiology and Preventive Medicine, School of Public Health, Sackler Faculty of Medicine, Tel-Aviv University, Tel-Aviv 6997801, Israel; 4 The Edmond and Lily Safra Children Hospital, Sheba Medical Center, Ramat Gan 52621, Israel; 5 Department of Pediatrics, Shamir Medical Center, Zeriffin 703301, Israel

**Keywords:** trophic feeding, breastmilk, very low birth weight, formula feeding

## Abstract

Aim: To consider the question of whether to initiate trophic feeds with formula in the absence of own mother’s breastmilk or to wait for breastmilk to be available. Methods: A retrospective study of infants born prior to 32 weeks of gestation during the period 2012–2017 at a single tertiary center in Tel Aviv, Israel. Three TF groups were defined: exclusive breastmilk, mixed, and exclusive formula. Univariate and multivariate analyses were conducted. Logistic regression was used, and adjusted odds ratio and 95% interval were reported. Results: Univariate analysis demonstrated that infants in the exclusive breastmilk group were born earlier, had lower birth weights and lower Apgar scores, were given lower volumes of TF, and were more likely to have a longer hospital stay. Poor composite outcome was more common among the exclusive breastmilk group. Multivariate regression analysis revealed no differences in incidence of early neonatal morbidities between the groups, except for longer duration of parenteral nutrition in the exclusive breastmilk group. Conclusion: In our cohort, exclusive formula TF was not associated with increased risk of any of the studied morbidities. Clinicians should consider this finding in deciding between early TF or fasting while waiting for own mother’s breastmilk.

## 1. Introduction

Adequate nutrition is essential for the optimal growth and development of infants born prematurely. Preterm birth is associated with increased risk of feeding intolerance and necrotizing enterocolitis (NEC). NEC incidence is inversely related to gestational age, with 90% of cases occurring after infants have been fed [1]. On the other hand, withholding enteral nutrition necessitates prolonged use of indwelling vascular catheters and total parenteral nutrition, which are associated with increased risk of sepsis, cholestasis, longer time needed to achieve full enteral feeding and prolonged hospital stay [2,3]. Hence, the issue of how to achieve full enteral feeding within the shortest possible time while maintaining optimal nutritional needs and avoiding complications is challenging.

Over the last two decades, early initiation of non-nutritive feeding, also referred to as trophic feeding (TF), has become common. Trophic feeding refers to the introduction of minimal volumes of enteral nutrition, up to 24 mL/kg/day for the first several days of life. Trophic feeding is administered not as a source of nutrition but rather for its positive effect on the gut [4,5]. Trophic feeding promotes intestinal motility as well as the secretion of enzymes and trophic hormones necessary for digestion [4,6,7]. In a cohort study of 170 very low birth weight infants, TF resulted in reduced duration of parenteral nutrition, improved tolerance of nutritive enteral feeds, increased weight gain during the first days of life, and reduced hospital stay [8]. Trophic feeding was shown to reduce the risk of sepsis among preterm infants, probably by maintaining the intestinal barrier which inhibits bacterial translocation [9].

Breastmilk, the recommended enteral nutrition for preterm infants [10], is associated with improved intestinal development, reduced risk of NEC, better neurodevelopmental outcomes and decreased risk of later metabolic and cardiovascular disease [10]. Breastmilk contains numerous bioactive factors, including antioxidants, growth factors, adipokines and cytokines, as well as unique nutritional factors such as fatty acids and fatty acid-derived terminal mediators that serve as an energy source and as regulators of development, immune function, and metabolism [11,12,13,14,15].

To the best of our knowledge, the effect of TF content on early neonatal outcomes has not yet been studied. Our primary objective was to assess the effect of TF content on early gastrointestinal morbidities. We also sought to assess the effect of TF content on other common early neonatal morbidities, such as late onset sepsis, duration of parenteral nutrition and death. We hypothesized that TF content would not have a negative impact on early neonatal outcomes among preterm infants.

## 2. Methods

### 2.1. Study Design

**Figure 1:**
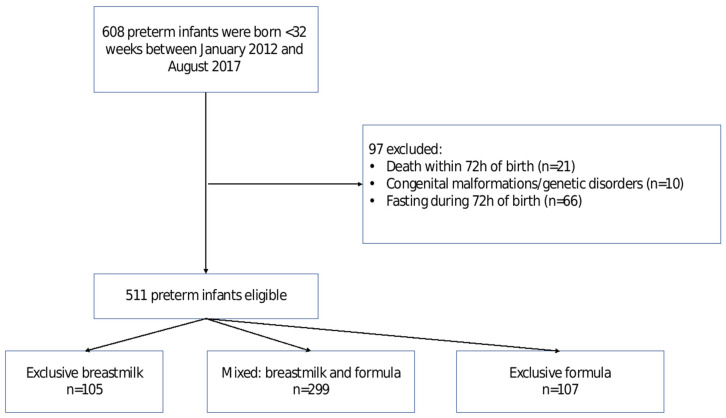
Flow chart of infants in the study.

Infants born <32 weeks of gestation during the period 2012–2017 at a single tertiary medical center were eligible for the study. Trophic feeding was defined as any enteral nutrition ≤24 mL/kg/day introduced within the first 72 h of life. Infants with major congenital anomalies or genetic syndromes or those who died within the first 72 h of life were excluded. In addition, infants who did not receive TF were excluded, as they represent a different and sicker group of infants. During the study period, 608 preterm infants were born at the medical center. Of these, 97 infants were excluded (21 died within the first 72 h of life, 10 were diagnosed with genetic abnormalities, 66 did not receive TF) (Figure 1). Hence, the final cohort included 511 infants.

### 2.2. Data Collection

Perinatal data were collected retrospectively from the computerized system of the neonatal department (MetaVision, iMD soft, Dusseldorf, Germany). These data included maternal and infant characteristics such as maternal hypertension (defined as any hypertension diagnosed during pregnancy), clinical chorioamnionitis (defined as maternal fever accompanied by fetal tachycardia, foul smelling amniotic fluid and abdominal tenderness), antenatal steroids (partial or complete course), multiple pregnancy, gestational age (GA), birth weight (BW), BW z-score, neonatal sex, and Apgar scores at 1 and 5 min. Gestational age was determined using first trimester ultrasound and/or the date of the mother’s last menstrual period. BW z-score was measured according to the Fenton growth curves [16].

Trophic feeding content (breastmilk vs. formula) and volume during the first 72 h of life were documented. Three groups of TF were defined: exclusive breastmilk group (100% breastmilk), exclusive formula group (100% formula) and mixed group (breastmilk and formula).

The study examined the following outcomes: duration of oxygen requirement, bronchopulmonary dysplasia (BPD) defined as oxygen treatment at 36 weeks, duration of total parenteral nutrition, late onset sepsis (beyond the first 72 h of life) [17], spontaneous intestinal perforation (SIP), NEC (Bell’s stage ≥ II), [18] bloody stools, and death prior to discharge.

The following outcomes were skewed: duration of total parenteral nutrition, oxygen requirement, and hospital stay. Hence, we transformed these outcomes into categorical variables by defining them as “longer” if their levels were above the 66th percentile of the cohort.

Because we could not exclude unfavorable outcomes (e.g., BPD, longer oxygen requirement) in infants who died prior to discharge, for the purpose of statistical analysis, unfavorable outcomes and death were calculated as a composite of the prespecified outcome. Poor composite outcome was defined as “yes” if any of the following occurred: NEC, SIP, bloody stool, sepsis, BPD or death.

### 2.3. Nutrition Protocol

During the study period, the nutritional guidelines at our institution included early (i.e., begun once intravenous access was available) parenteral nutrition using 1.5–2 g/kg/day amino acids and 0.5 g/kg/day lipids. Proteins and lipids were increased gradually by 1 g/kg/day, up to 3 g/kg/day as tolerated. Mothers were strongly encouraged to express breastmilk soon after birth. Trophic feeding was initiated on the first day of life, usually within 6 h after birth, with preference given to breastmilk, when available. During the study period, donor breastmilk was not available in Israel. If breastmilk was not available, preterm infant formula (Materna Pagim, Nestle, Kibutz Ma’abarot, Irael) was given, except in the case of infants whose parents requested exclusive breastmilk. Residuals were measured prior to each feeding. If residuals exceeded 50% of the previous bolus or were bilious, the following feed was skipped. If enteral nutrition was tolerated, after 48–72 h it was increased by increments of 20 mL/kg/day. At the same time, parenteral nutrition was gradually reduced to maintain the amount of daily fluid intake within the unit’s protocol. Parenteral nutrition was usually withheld upon reaching total fluid intake of 100–130 mL/kg/day, depending on the infant’s weight and at the discretion of the attending physician.

The study protocol was approved by the institutional committee on human research (5693-18-SMC). The committee waived the need for consent. The authors do not have any conflict of interest.

### 2.4. Statistics

Categorical variables were reported as numbers and percentages. Continuous variables were evaluated for normal distribution using histogram and Q-Q plot. Variables that were normally distributed were reported as means and SDs, whereas skewed variables were reported as medians and interquartile ranges. Chi square testing and Fisher’s Exact test were applied to study the association between the categorical variables. Independent samples *t*-tests were used to compare normally distributed continuous variables between categories, and Mann–Whitney tests were used to compare non-normal distributed.

Variables that exhibited significant associations with the study variables (exposure variables or outcome variables) and variables that were previously shown to affect outcomes were included in the multivariate analysis. Logistic regression was applied, and adjusted odds ratio and 95% interval were reported. All statistical tests were 2-sided, and *p* < 0.05 was considered to be statistically significant throughout the study. SPSS software was used for the analyses (IBM SPSS statistics for Windows, version 24, IBM corp., Armnok, NY, USA, 2015).

## 3. Results

A comparison between the 66 infants who did not receive TF and the study infants who received TF revealed that those in the “non-exposed” group were born significantly earlier, had lower BWs and lower BW z-scores, and were more likely to be born via cesarean section (Table 1). The final cohort (*n* = 511) was born at a median gestational age of 30.1 (28.6–31.1) weeks with a mean BW of 1242 ± 320 gr. Median duration of parenteral nutrition was 9 (6–13) days. Bloody stools occurred in 36 (7.0%), NEC in 15 (3.1%), and sepsis in 115 (22.9%). Twelve infants (2.3%) died, and median age at death was 13 (9.5–22) days. Table 2 shows the clinical characteristics and early neonatal morbidities of the entire study cohort. In accordance with the predefined criteria (see Methods), prolonged oxygen treatment was defined as ≥15 days, prolonged parenteral nutrition was defined as ≥12 days and prolonged hospital stay was defined as ≥37.4 weeks post menstrual age (PMA). The participating infants were divided into three groups according to TF content: exclusive breastmilk; *n* = 105 (20.5%), exclusive formula; *n* = 107 (20.9%); and mixed (combination of breastmilk and formula) *n* = 299 (58.5%). Three comparisons were conducted: exclusive breastmilk vs. any formula (mixed + exclusive formula); exclusive formula vs. any breastmilk (mixed + exclusive breastmilk); exclusive breastmilk vs. exclusive formula (Table 3). Univariate analysis revealed that those in the exclusive breastmilk group were born significantly earlier (29 vs. 30.4 vs. 30.4 weeks, *p* < 0.001), had lower BWs (1072 vs. 1286 vs. 1293 gr, *p* < 0.001), had lower Apgar scores, and were given lower volumes of TF (3.2 vs. 10.9 vs. 10.4 mL/kg/day, *p* < 0.001) compared to those fed any formula and those fed exclusive formula, respectively.

In terms of early neonatal morbidities, those in the exclusive breastmilk group were more likely to be diagnosed with BPD (20% vs. 7.4% vs. 5.6% *p* < 0.001 and *p* = 0.002), require longer oxygen and intravenous fluid treatment, and stay in the hospital longer than those fed any formula and those fed exclusive formula, respectively.

Poor composite outcome (defined as any of the following: NEC, SIP, bloody stool, sepsis, BPD or death) was significantly more common among the exclusive breastmilk fed group than among the any formula or the exclusive formula group, respectively (61.9% vs. 37.2% vs. 35.5% *p* < 0.001).

Outcomes did not differ significantly between the exclusive formula group and the any breastmilk (exclusive breastmilk + mixed) group. Occurrence of NEC, bloody stool or sepsis did not differ significantly between the groups.

**Figure 2:**
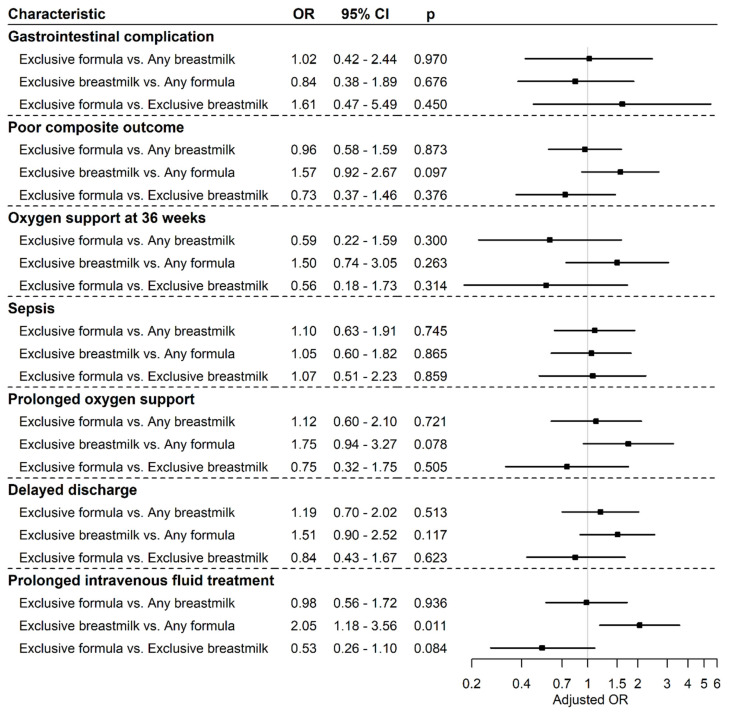
Multivariate analysis, forest plot. (Israel, 2012–2017).

Multivariate analysis controlling for gender, GA, BW z-score, chorioamnionitis, antenatal steroids exposure, ibuprofen treatment, and 5 min Apgar score revealed no differences in early neonatal outcomes between the groups, with the exception of longer intravenous fluid treatment in the exclusive breastmilk group (Figure 2).

## 4. Discussion

In this retrospective study of more than 500 infants born at <32 weeks of gestation, we assessed the effect of TF content on early neonatal outcomes. Nearly two-thirds of the cohort received mixed TF (breastmilk and formula), whereas the rest received either exclusive breastmilk (20.5%) or exclusive formula (20.9%). The infants in the exclusive breastmilk group differed significantly from those in other groups in that they were born earlier, had lower birth weights, and had lower Apgar scores. This group also received a significantly smaller amount of TF during the first 72 h of life. As expected, this group was more likely to be diagnosed with BPD, poor composite outcome, longer hospital stays and longer duration of intravenous fluid treatment. Nevertheless, the incidence of gastrointestinal complications or sepsis did not differ between the groups. Multivariate analysis correcting for gender, GA, BW-z score, amnionitis, ibuprofen treatment and exposure to antenatal steroids did not reveal any differences in early neonatal morbidities, with the exception of longer duration of parenteral nutrition among the exclusive breastmilk group. Specifically, the exclusive formula group was not shown to be at increased risk of any of the studied morbidities. Therefore, we concluded that in the present cohort, the content of TF given during the first 72 h of life did not affect the risk of early neonatal morbidities or mortality.

Early TF and breastmilk nutrition are currently the standard of care in neonatology. Both have been shown to have a positive effect in reducing common morbidities [10,19]. Difficulties in breastmilk production are common among mothers of preterm infants, especially during the first days after giving birth, and may affect the availability of own mothers’ milk for early enteral TF [20]. In some countries, TF using donor milk may be offered as a bridge until own mothers’ milk is available [21]. Yet, in countries in which donor milk is not available, clinicians must decide whether to expose infants to formula or to withhold enteral feeding until breastmilk is available, thus prolonging the duration of intravenous parenteral nutrition and its associated risks. To the best of our knowledge, no studies to date have evaluated the significance of TF content on early neonatal outcomes.

In the present study, formula TF was not associated with increased risk of any of the studied morbidities. A study conducted in England examined the effect of exclusive breastmilk during the first three days of life in decreasing the risk of severe NEC among a large cohort of infants born prior to 32 weeks GA. In that study, a high number of infants (114) needed to be exclusively breastfed in order to prevent one case of severe NEC (95% CI 87 to 136), such that the absolute risk reduction by exclusive breastmilk was less than 1% [22]. In addition, a recent Cochrane review that compared the use of formula vs. donor breastmilk for feeding preterm or low birth weight infants found no major differences in outcomes [21].

The current study also assessed symptoms such as bloody stool and SIP that may represent a milder form of NEC. The results did not show any significant differences between the groups. Even though one may argue that the formula fed group was at lower risk because it was more mature, these results persisted even after correcting for various confounders.

Among infants exposed to exclusive breastmilk, TF volumes were significantly lower than in the others two groups. We speculate that the lower volumes of TF in this group may be due to reluctance among the medical staff to introduce formula feeds to the smallest preterm infants. It is also possible that this finding is secondary to mothers’ greater commitment to supply exclusive breastmilk to the smallest and sickest infants, as shown in our previous study [23]. It is interesting to note that although these infants required longer intravenous fluid treatment, they did not demonstrate an increased rate of sepsis. Here, we speculate that the protective effect is associated with the actual exposure to TF and is less affected by TF volume. This conjecture is supported by previous studies demonstrating a protective effect of low volumes of TF (10 mL/kg/day), even among infants with feeding intolerance [19]. Infants who were not exposed to TF were excluded from that study, based on our hypothesis that they represent a different and sicker group of infants. In light of the current data, we believe that even the smallest and sickest infants should be given TF.

This study has several limitations, among them its retrospective nature and the low incidence of adverse outcomes, necessitating the creation of a composite parameter of poor outcome. Moreover, the study did not assess maternal dietary patterns or maternal body composition, which have been shown to influence breastmilk compounds and newborns’ health. [24] On the other hand, the study also has several strengths, including a large sample of infants born at a single center with a uniform feeding protocol and the unequivocal results of the multivariate analysis, as demonstrated in the forest plot (Figure 2).

In conclusion, when clinicians are faced with the dilemma of whether “to feed or not to feed” in the absence of breastmilk, our data support the administration of early TF with formula. The results show that TF with formula was not associated with an increased risk of any of the studied adverse outcomes in the present cohort of infants born at <32 weeks of gestation. This finding is important for resource-poor countries in which pasteurized donor human milk is not an option. Further research involving randomized or case–control studies is needed to evaluate the effect of TF content on neonatal outcomes among infants who are extremely premature, small for gestational age and have very low birth weights. Further studies should also address the role of donor milk TF in infants born at <32 weeks of gestation.

## Figures and Tables

**Table 1:** Comparison of infants exposed to trophic feeding and those not exposed to trophic feeding. (Israel, 2012–2017).

	Trophic Feeding-Yes (*n* = 511)	Trophic Feeding-No (*n* = 66)	*p*-Value
Birth weight (grams)	1242 (320)	941 (361)	<0.001
Gestational age (weeks)	30.1 (28.6–31.1)	28.1 (25.9–30.5)	<0.001
Birth weight Z-score	−0.2 (0.7)	−0.6 (1.1)	0.01
Gender (female)	263 (51.5%)	29 (43.9%)	0.25
Multiple birth	281 (55%)	30 (45%)	0.14
Antenatal steroids	474/508 (93.3%)	57/65 (87.7%)	0.12
Surgical delivery	359 (70.3%)	56 (84.8%)	0.013
Amnionitis	43 (8.4%)	5/65 (7.7%)	0.84
Maternal hypertension	64 (12.5%)	6 (9.1%)	0.42

Data are shown as mean (SD), median (interquartile) or number (%).

**Table 2:** Clinical characteristics of included infants. (Israel, 2012–2017).

	Studied Infants (*n* = 511)
Birth weight (gram)	1242 (320)
Gestational age (weeks)	30.1 (28.6–31.1)
Birth weight Z-score	−0.2 (0.7)
Gender (female)	263 (51.5%)
Multiple birth	281 (55%)
Clinical amnionitis	43 (8.4%)
Maternal hypertension	64 (12.5%)
Antenatal steroids	474/508 (93.3%)
Surgical delivery	359 (70.3%)
Apgar 1′	8 (6–9)
Apgar 5′	9 (9–10)
Necrotizing enterocolitis	15 (3.1%)
Age at necrotizing enterocolitis (days)	17 (12.5–38.7)
Spontaneous intestinal perforation (SIP)	4 (0.8%)
Age at spontaneous intestinal perforation (days)	6.5 (3.75–7.7)
Bloody stool	36 (7%)
Age at Bloody stool	15 (12–28.3)
Ibuprofen treated patent ductus arteriosus	70 (13.7%)
Late onset sepsis	115 (22.9%)
Age at late onset sepsis (days)	9 (7.1–13.5)
Oxygen treatment at week 36 (BPD)	39 (7.6%)
Duration of oxygen treatment (days)	6 (1–21)
Intravenous fluid treatment days	9 (6–13)
Enteral feeding during first 72 h (ml/kg/day)	7.75 (3.8–15.9)
Intraventricular hemorrhage grade ≥ 3	19/510 (3.8%)
Gestational age at live discharge	36.71 (36–37.9)
Death	12 (2.3%)
Age at death (days)	13 (9.5–21.2)

Data are presented as mean ± SD, number (%) or median (interquartile).

**Table 3:** Demographic and clinical characteristics of breastmilk fed infants vs. others. (Israel, 2012–2017).

	Exclusive Formula (*n* = 107)	Any Breastmilk (*n* = 404)	*p*-Value	Exclusive Breastmilk (*n* = 105)	Any Formula (*n* = 406)	*p*-Value	Exclusive Breastmilk (*n* = 105)	Exclusive Formula(*n* = 107)	*p*-Value
Birth weight (gr)	1293 (282)	1229 (328)	0.045	1072 (318)	1286 (306)	<0.001	1072 (318)	1293 (282)	<0.001
Gestational age (weeks)	30.4 (29–31.3)	30.1 (28.3–31.1)	0.05	29 (26.7–30.7)	30.4 (28.9–31.3)	<0.001	29 (26.7–30.7)	30.4 (29–31.3)	<0.001
Birth weight Z-score	−0.2 (0.7)	−0.2 (0.8)	0.59	−0.3 (0.8)	−0.2 (0.7)	0.27	−0.3 (0.8)	−0.2 (0.7)	0.31
Gender (female)	60 (56.1%)	203 (50.2%)	0.28	54 (51.4%)	209 (51.5%)	0.99	54 (51.4%)	60 (56.1%)	0.49
Multiple birth	63 (58.9%)	218 (54%)	0.36	56 (53.3%)	225 (55.4%)	0.70	56 (53.3%)	63 (58.9%)	0.42
Clinical amnionitis	14 (13.1%)	29 (7.2%)	0.05	8 (7.6%)	35 (8.6%)	0.74	8 (7.6%)	14 (13.1%)	0.19
Maternal hypertension	13 (12.1%)	51 (12.6%)	0.89	14 (13.3%)	50 (12.3%)	0.78	14 (13.3%)	13 (12.1%)	0.79
Antenatal steroids	104/105 (99%)	370/403 (91.8%)	0.008	95 (90.5%)	379/403 (94%)	0.19	95 (90.5%)	104/105 (99%)	0.005
Surgical delivery	79 (73.8%)	280 (69.3%)	0.36	77 (73.3%)	282 (69.5%)	0.44	77 (73.3%)	79 (73.8%)	0.93
Apgar 1′	8 (6–9)	8 (6–9)	0.21	7 (5–9)	8 (6–9)	<0.001	7 (5–9)	8 (6–9)	0.001
Apgar 5′	9 (9–10)	9 (9–10)	0.89	9 (8–10)	9 (9–10)	<0.001	9 (8–10)	9 (9–10)	0.008
Enteral feeding during first 72 h (ml/kg/day)	10.4 (3.9–16.5)	7.2 (3.8–15.5)	0.25	3.2 (1.5–5.6)	10.9 (5–17.6)	<0.001	3.2 (1.5–5.6)	10.4 (3.9–16.5)	<0.001
Necrotizing enterocolitis	4 (3.7%)	12 (3%)	0.75	5 (4.8%)	11 (2.7%)	0.34	5 (4.8%)	4 (3.7%)	0.75
SIP	0 (0%)	4 (1%)	0.58	3 (2.9%)	1 (0.2%)	0.03	3 (2.9%)	0 (0%)	0.12
Bloody stool	6 (5.6%)	30 (7.4%)	0.51	8 (7.6%)	28 (6.9%)	0.79	8 (7.6%)	6 (5.6%)	0.55
Gastrointestinal complications	7 (6.5%)	35 (8.7%)	0.48	10 (9.5%)	32 (7.9%)	0.58	10 (9.5%)	7 (6.5%)	0.42
Sepsis	23 (21.5%)	92/395 (23.3%)	0.69	29/101 (28.7%)	86/401 (21.4%)	0.12	29/101 (28.7%)	23 (21.5%)	0.23
Death	2 (1.9%)	10 (2.5%)	>0.999	2 (1.9%)	10 (2.5%)	>0.999	2 (1.9%)	2 (1.9%)	>0.999
Oxygen treatment at week 36 (BPD)	6 (5.6%)	45 (11.1%)	0.09	21 (20%)	30 (7.4%)	<0.001	21 (20%)	6 (5.6%)	0.002
Poor composite outcomes	38 (35.5%)	178 (44.1%)	0.11	65 (61.9%)	151 (37.2%)	<0.001	65 (61.9%)	38 (35.5%)	<0.001
Prolonged oxygen support (>15 days)	31 (29%)	143 (35.7%)	0.19	60 (57.7%)	114 (28.2%)	<0.001	60 (57.7%)	31 (29%)	<0.001
Delayed discharge (>37.44 weeks)	32 (30.2%)	135 (33.8%)	0.49	52 (50.5%)	115 (28.5%)	<0.001	52 (50.5%)	32 (30.2%)	0.003
Prolonged intravenous fluid treatment (>12 days)	30 (28%)	148 (36.9%)	0.09	61 (58.7%)	117 (29%)	<0.001	61 (58.7%)	30 (28%)	<0.001

Data are presented as mean ± SD, number (%) or median (interquartile). SIP—spontaneous intestinal perforation, BPD—bronchopulmonary dysplasia.

## Data Availability

Not applicable.

## Abbreviations

bronchopulmonary dysplasia (BPD), birth weight (BW), gestational age (GA), intravenous (IV), necrotizing enterocolitis (NEC), post menstrual age (PMA), spontaneous intestinal perforation (SIP), trophic feeding (TF)
